# Development of an immune-related gene pairs index for the prognosis analysis of metastatic melanoma

**DOI:** 10.1038/s41598-020-80858-1

**Published:** 2021-01-13

**Authors:** Rong-zhi Huang, Min Mao, Jie Zheng, Hai-qi Liang, Feng-ling Liu, Gui-you Zhou, Yao-qing Huang, Fan-yue Zeng, Xu Li

**Affiliations:** 1 Second Department of Orthopaedics, The First People’s Hospital of Qinzhou, No.8, Mingyang Road, Qinzhou, 535000 The Guangxi Zhuang Autonomous Region China; 2 Guangxi Medical University, Nanning, 530021 The Guangxi Zhuang Autonomous Region China; 3 Thyroid Gland Breast Surgery, The First People’s Hospital of Qinzhou, Qinzhou, 535000 The Guangxi Zhuang Autonomous Region China

**Keywords:** Tumour biomarkers, Skin cancer, Melanoma, Data mining, Cancer, Computational biology and bioinformatics, Biomarkers

## Abstract

Melanoma is a skin cancer with great metastatic potential, which is responsible for the major deaths in skin cancer. Although the prognosis of melanoma patients has been improved with the comprehensive treatment, for patients with metastasis, the complexity and heterogeneity of diffuse diseases make prognosis prediction and systematic treatment difficult and ineffective. Therefore, we established a novel personalized immune-related gene pairs index (IRGPI) to predict the prognosis of patients with metastatic melanoma, which was conducive to provide new insights into clinical decision-making and prognostic monitoring for metastatic melanoma. Through complex analysis and filtering, we identified 24 immune-related gene pairs to build the model and obtained the optimal cut-off value from receiver operating characteristic curves, which divided the patients into high and low immune-risk groups. Meantime, the Kaplan–Meier analysis, Cox regression analysis and subgroup analysis showed that IRGPI had excellent prognostic value. Furthermore, IRGPI was shown that was closely associated with immune system in the subsequent tumor microenvironment analysis and gene set enrichment analysis. In addition, we broken through the data processing limitations of traditional researches in different platforms through the application of gene pairs, which would provide great credibility for our model. We believe that our research would provide a new perspective for clinical decision-making and prognostic monitoring in metastatic melanoma.

## Introduction

Melanoma is one of the most immunogenic tumors due to its high genomic mutational load^1,2^. It accounts for only two percent of all skin cancers but causes the most deaths^3^. Through the combination of treatments, the 5-years relative survival rate for persons with melanoma is good, at 92%^4^. However, there are still a small number of patients with poor prognosis due to the propensity of melanoma to spread^5^. The prognosis prediction and systemic treatment are difficult and not always productive in diffusion disease due to its complexity and heterogeneity^1,6,7^. Accordingly, it is urgent to find out novel personalized therapeutic strategies and biomarkers to improve and monitor the unfavourable prognosis of metastatic melanoma.

Growing studies reveals that the immune system plays a key role in the development and progression of cancers and the relationship between body immune and tumor is complicated^8–10^. Tumor cells express certain antigenic components, and immune cells can penetrate into tumor tissues through chemotaxis for immune clearance^8,11^. However, when tumor microenvironment is disordered, tumor cells would evade immune elimination and suppress immune response, thus leading to tumor progression^12,13^. In melanoma, existed studies have also shown that the initiation and progression of melanoma are closely related to tumor immunity^8,12^. What's more, immunotherapy in melanoma has been widely studied on this basis^14–16^.

ENK et al. applied interleukin-2 (IL-2) inhalation therapy to patients with pulmonary metastatic melanoma by transferring cytokines to the tumor site, which reduced the toxicity associated with systemic IL-2 administration and achieved good efficacy^17^. Interleukin-21 (IL-21) was mainly produced by T helper cell 17 (Th17) and played a key role in the development of Th17. It had strong anti-tumor activity^18^. Petrella et al. proved the activity of IL-21 in metastatic melanoma through a multicenter phase II study, and it had a certain curative effect on metastatic melanoma^19^. High expression of cytotoxic T-lymphocyte-associated protein 4 (CTLA-4) was closely related to antigen-specific T cell dysfunction in metastatic melanoma. Relevant preclinical studies showed that the introduction of inhibitory antibodies to CTLA-4 could eliminate downstream inhibitory signals, thus avoiding the dysfunction of antigen-specific T cell, which could produce a cytotoxic anti-tumor response^20,21^. Meanwhile, a clinical trial in the antitumor activity of programmed cell death ligand 1/programmed cell death 1(PD-L1/PD-1) signaling blocking was confirmed useful in multiple types of cancers, including advanced melanoma^22^. These studies proved that tumor immunity was closely related to the progression and treatment of metastatic melanoma, so a new personalized comprehensive prognosis index based on immunity would be very promising.

In our study, we attempt to establish a novel prognosis index to predict the prognosis of patients with metastatic melanoma, which is based on the screening of gene pairs and could greatly reduce the biological heterogeneity and technical bias of different cross-sequencing platforms. We hope to provide a novel insight for prognosis prediction and clinical decision-making for metastatic melanoma patients.

## Materials and methods

### Data acquisition

**Figure 1:**
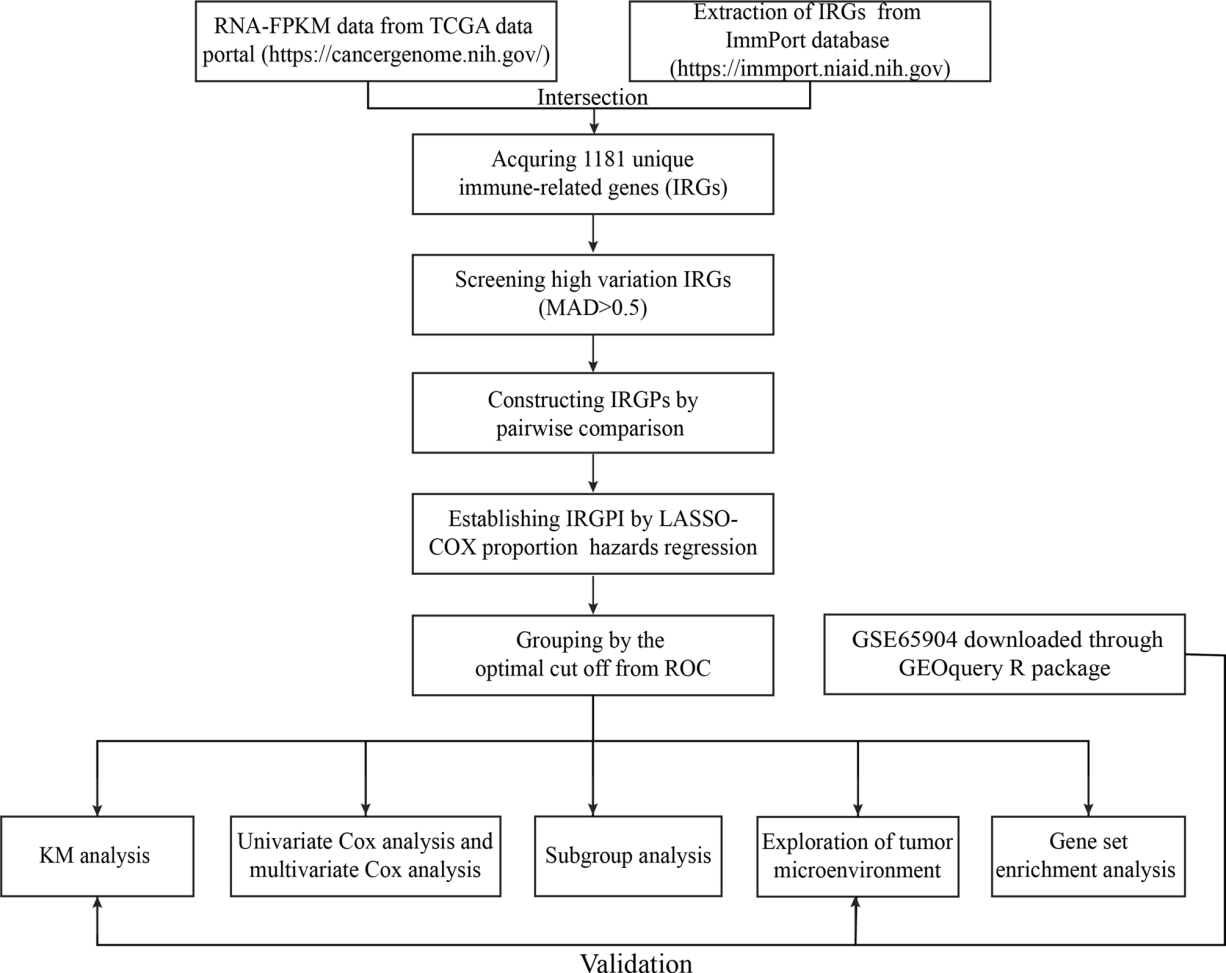
The flowchart of IRGPI establishment and validation.

The flowchart of IRGPI establishment and validation is presented in Fig. 1. The fragments per kilobase of exon model per million mapped reads (FPKM) RNA- seq data and clinical data of melanoma samples were obtained from The Cancer Genome Atlas (TCGA) data portal (https://cancergenome.nih.gov/). The samples with no metastatic foci, no follow-up information, or follow-up time less than 30 days were excluded. Then, 354 metastatic melanoma samples were reserved for training dataset. Additionally, the expression profiles of GSE65904 were downloaded for testing dataset through GEOquery R package. The same sample filtering standard was used for GSE65904 dataset and 186 metastatic melanoma samples were retained. Finally, 1811 unique immune-related genes (IRGs) were acquired from ImmPort database (https://immport.niaid.nih.gov) to construct immune-related prognostic signature.

### Data preprocessing

The Ensembl IDs of RNA-FPKM data were transformed into gene symbols based on the Ensembl database (http://asia.ensembl.org/index.html). The probe IDs of GSE65904 expression profiles were converted into gene symbols through the illuminaHumanv4.db R package. Ensembl IDs or probe IDs were retained on basis of the mean overall expression of each gene. Next, only IRGs measured by all platforms with relatively high variation (determined by MAD > 0.5, MAD: median absolute deviation) were selected for further analysis.

### Establishment of immune-related gene pairs index for prognosis prediction

The immune-related gene pairs (IRGPs) were constructed by pairwise comparison of the gene expression level in a specific sample or profile. If the expression level of the first IRG was higher than that of the second IRG, the score of this IRGP was 1; otherwise, the score was 0. Next, the establishment of immune-related gene pairs index (IRGPI) was based on previous description^23^. The RNA-FPKM data was identified as training dataset for establishing the IRGPI through using Lasso Cox proportion hazards regression with tenfold cross validation (glmnet R package, version: 3.0-2). The IRGPs of training dataset with a small variation and imbalanced distribution (MAD = 0) were excluded from the analysis. Then, we conducted 1000 times Lasso Cox proportion hazards regression analysis into training dataset, in which the model with the most occurrences was identified as the most stable gene pairs model and was used for the development of IRGPI.

### Kaplan–Meier curve analysis and validation of IRGPI

The prognosis risk of metastatic melanoma patients was distinguished based on IRGPI and the time-dependent receiver operating characteristic (ROC) curve analysis within five years was performed to determine the optimal cut off in the training dataset. KM curve analysis and subgroup analysis were further used to evaluate the ability of IRGPI to distinguish survival risk of metastatic melanoma. Univariate and multivariate Cox analyses were conducted to compare the survival impact of IRGPI with other clinical characteristics. Further, the prognosis ability of IRGPI was validated in the independent GSE65904 testing cohort by KM curve analysis.

### Tumor microenvironment in different IRGPI risk groups

The underlying tumor microenvironment mechanisms of different survival prediction impact of IRGPI on metastatic melanoma were explored by the single-sample gene-set enrichment analysis (ssGSEA). It quantified 29 tumor microenvironment immune cell infiltrating score in each sample by using 29 immune gene sets^24^. The immune score of each sample was calculated by ESTIMATE R package^9^. We used the t test to identify the difference of tumor immune microenvironment between the high and low-IRGPI risk groups.

### Immune related biological processes in different IRGPI risk groups

The ordered gene lists of two cohorts were identified through corresponding R packages (training cohort: edgeR R package, testing cohort: limma R package) and Gene Set Enrichment Analysis (GSEA)^25^ was conducted on the gene with false discovery rate (FDR) less than 0.05 to identify biological processes that were differently activated between the high and low-IRGPI risk groups. The number of random sample permutations were set at 1000 and the min size of gene set was set at 160. The biological processes with FDR < 0.01 in both cohorts indicated a statistically significant difference.

### Statistical analysis

All statistical analyses were conducted using the R software (version 3.6.3) (http://www.r-project.org/) and its corresponding R packages. The Area Under Curve (AUC) of ROC curve was performed using the survialROC R package. KM curve analysis was completed using log-rank test from survminer R package. GSEA analysis was completed using clusterProfiler R package. The c5.bp.v7.1.entrez.gmt file from the Molecular Signatures Database (MSigDB, http://software.broadinstitute.org /gsea/index.jsp) was obtained for the GSEA to identify biological processes. All P value of less than 0.05 indicated a statistically significant difference in all analysis.

## Results

### Establishment of immune-related gene pair index for prognosis prediction

**Figure 2:**
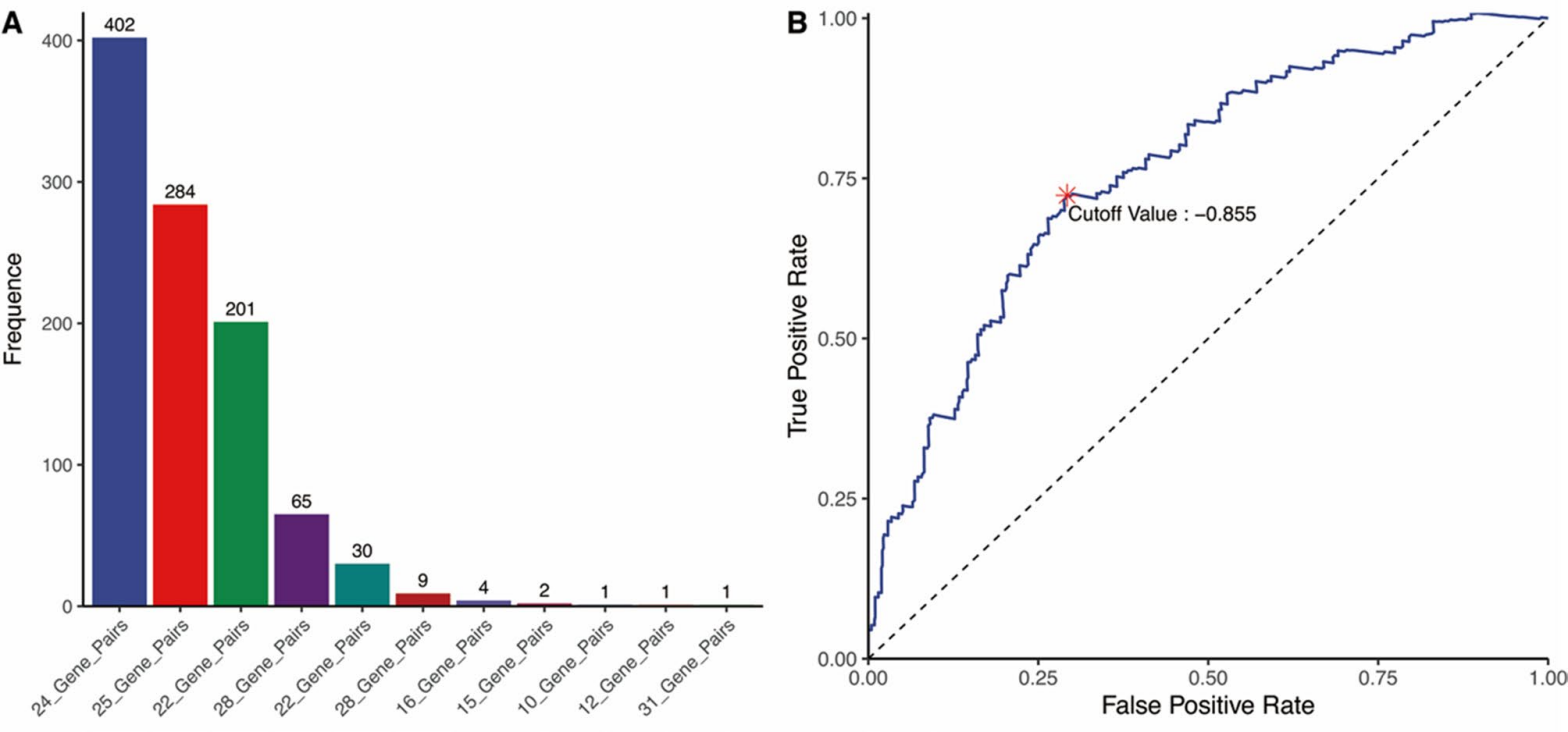
(**A**) The count of model of 1000 times Lasso Cox proportion hazards regression into training set. The 24 IRGPs with the highest frequency were selected for the development of IRGPI. (**B**) Time dependent receiver operating characteristic (ROC) curve within five years for IRGPI in the training cohort. The optimal IRGPI of − 0.855 was performed as cut off to divided patients into low- or high-IRGPI risk groups.

In the analysis, 376 IRGs of 354 TCGA metastatic melanoma samples were retained for constructing the IRGPs. The detail clinical features of TCGA cohorts were shown in Table 1. After removing IRGPs with relatively small variation (MAD = 0), 119 IRGPs were left for initial candidate IRGPs. Next, 24 IRGPs with the highest frequency were selected for the development of IRGPI through the 1000 times Lasso Cox proportion hazards regression in training cohort (Fig. 2A). The information of 24 IRGPs and 45 unique IRGs from IRGPs was shown in Table 2.

**Table 1:** The detail clinical features and the results of univariate and multivariable Cox regression analyses in training cohort.

Level	Number (%)	Univariate cox analysis	Multivariate cox analysis	
		*p* Value	HR (95%CI)	*p* Value
**Age**				
< 60	202 (57.1)	3.15e−15*	10.09 (20.93–4.86)	5.5e−10*
≥ 60	152 (42.9)			
**Gender**				
Male	224 (63.3)	2.74e−04*	1.49 (2.13–1.04)	0.03*
Female	130 (36.7)			
**T stage**				
T0	23 (6.5)	0.271	1.01 (1.46–0.69)	0.97
T1	40 (11.3)			
T2	71 (20.1)			
T3	80 (22.6)			
T4	68 (19.2)			
Tis	7 (2.0)			
TX	42 (11.9)			
NA	23 (6.5)			
**N stage**				
N0	171 (48.3)	0.036*	1 (1.15–0.88)	0.97
N1	65 (18.4)			
N2	39 (11.0)			
N3	45 (12.7)			
NX	18 (5.1)			
NA	16 (4.5)			
**M stage**				
M0	313 (88.4)	0.005*	1.17 (1.37–0.99)	0.06
M1	19 (5.4)			
NA	22 (6.2)			
**Stage**				
I	74 (20.9)	0.359	1.26 (3.04–0.52)	0.61
II	59 (16.7)			
III	143 (40.4)			
IV	18 (5.1)			
NA	60 (16.9)			
**Radiation therapy**				
NO	264 (74.6)	5.42e−04*	1.25 (1.64–0.95)	0.11
YES	72 (20.3)			
NA	18 (5.1)			
**Disease free survival**				
NO TUMOR	132 (37.3)	0.003*	1.07 (1.61–0.71)	0.76
TUMOR	215 (60.7)			
NA	7 (2.0)			
**IRGPI**				
High IRGPI	192 (54.2)	0.714	2.32 (3.4–1.58)	1.9e−05*
Low IRGPI	162 (45.8)			
**OS status**				
Dead	186 (52.5)	–	–	–
Alive	168 (47.5)			

Abbreviation: *HR *hazard ratio, *CI* confidential interval, *IRGPI* immune-related gene pairs index, *OS* overall survival.

**Table 2:** The information of 24 IRGPs in the selected model.

IRG 1	Full name	IRG 2	Full name	Coefficient
CREB1	cAMP responsive element binding protein 1	TNFSF13B	Tumor necrosis factor superfamily member 13b	0.022
HLA-DOB	Major histocompatibility complex, class II, DO beta	CETP	Cholesteryl ester transfer protein	− 0.083
HLA-DQA1	Major histocompatibility complex, class II, DQ alpha 1	C3	Complement C3	− 0.061
HLA-DQA1	Major histocompatibility complex, class II, DQ alpha 1	NR2F6	Nuclear receptor subfamily 2 group F member 6	− 0.025
CXCL14	C-X-C motif chemokine ligand 14	CMTM8	CKLF like MARVEL transmembrane domain containing 8	0.029
CXCL11	C-X-C motif chemokine ligand 11	FABP7	Fatty acid binding protein 7	− 0.033
CXCL11	C-X-C motif chemokine ligand 11	TUBB3	Tubulin beta 3 class III	− 0.079
CXCL13	C-X-C motif chemokine ligand 13	SHC4	SHC adaptor protein 4	− 0.001
CCL13	C–C motif chemokine ligand 13	FABP4	Fatty acid binding protein 4	− 0.063
CCL8	C–C motif chemokine ligand 8	TUBB3	Tubulin beta 3 class III	− 0.51
SLC22A17	Solute carrier family 22 members 17	GNAI1	G protein subunit alpha i1	− 0.295
NOX4	NADPH oxidase 4	PRKCB	Protein kinase C beta	− 0.173
IDO1	Indoleamine 2,3-dioxygenase 1	CXCR3	C-X-C motif chemokine receptor 3	− 0.014
TNFSF10	Tumor necrosis factor superfamily member 10	PGF	Placental growth factor	− 0.094
IRF1	Interferon regulatory factor 1	PPP3CB	Protein phosphatase 3 catalytic subunit beta	− 0.009
ZYX	Zyxin	GPI	Glucose-6-phosphate isomerase	− 0.097
ITGAV	Integrin subunit alpha V	PIK3CD	Phosphatidylinositol-4,5-bisphosphate 3-kinase catalytic subunit delta	− 0.049
ABCC4	ATP binding cassette subfamily C member 4	VEGFC	Vascular endothelial growth factor C	− 0.006
CDH1	Cadherin 1	IL6ST	Interleukin 6 signal transducer	0.139
CD72	CD72 molecule	NR2F1	Nuclear receptor subfamily 2 group F member 1	− 0.122
SEMA3B	Semaphorin 3B	NR2F2	Nuclear receptor subfamily 2 group F member 2	− 0.133
SEMA3C	Semaphorin 3C	MET	MET proto-oncogene, receptor tyrosine kinase	− 0.136
TNC	Tenascin C	SORT1	Sortilin 1	− 0.096
DKK1	Dickkopf WNT signaling pathway inhibitor 1	ITK	IL2 inducible T-cell kinase	0.07

Abbreviation: *IRGPs* immune related gene pairs, *IRG1* immune related gene 1, *IRG2* immune related gene 2.

### Kaplan–Meier curve analysis and validation of IRGPI

**Figure 3:**
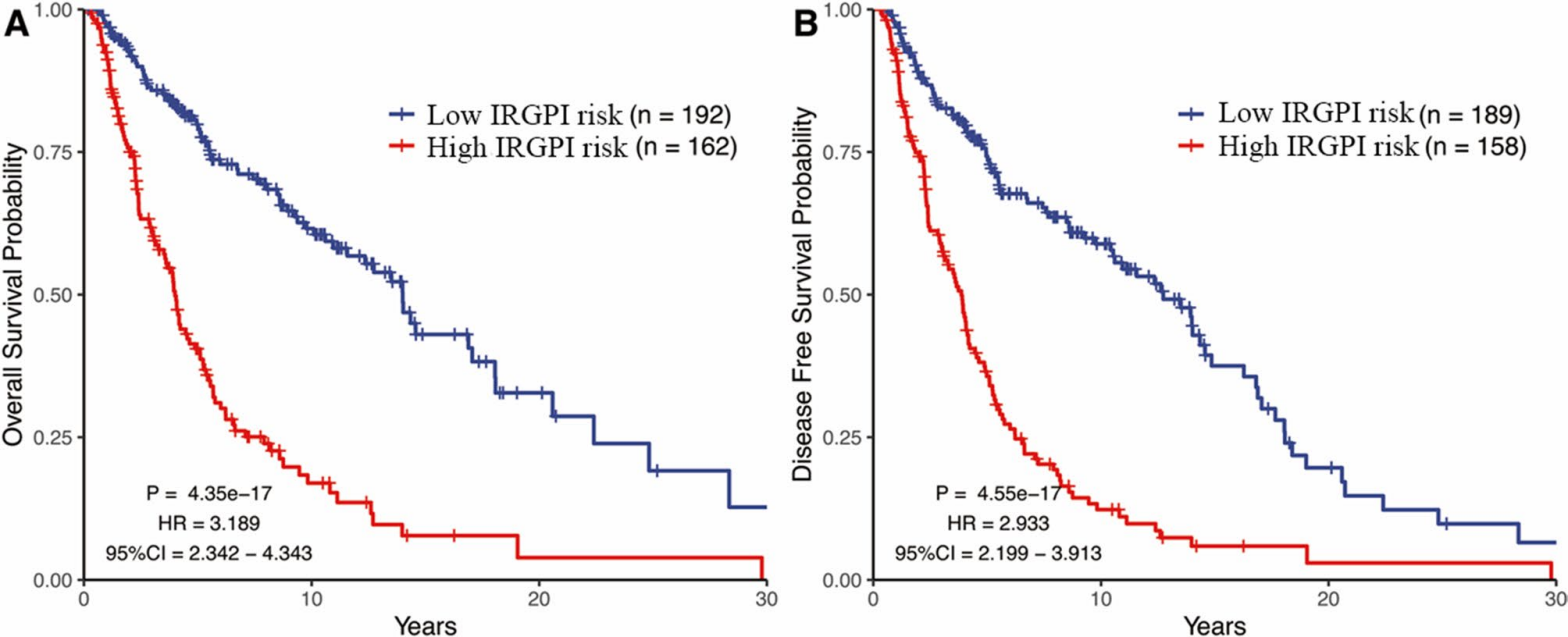
Kaplan**–**Meier curves analysis among different IRGPI risk groups in training cohort. (**A**) The impact of IRGPI on overall survival. (**B**) The impact of IRGPI on disease-free survival.

The optimal cut off of IRGPI for distinguishing patients into high and low-IRGPI risk group was identified as − 0.855 based on time-dependent ROC curve within five years (Fig. 2B). KM curve analysis indicated that patients with high-IRGPI risk were correlated with poorer overall survival (OS, HR: 3.189, 95%CI: 2.342–4.343, *p* = 4.35e−17) and disease-free survival (DFS, HR: 2.933, 95%CI: 2.199–3.913, p = 4.55e−17) in training cohort (Fig. 3). Furthermore, comparison with other clinical characteristic by univariate and multivariate Cox regression analyses showed that IRGPI maintained independently associated with OS (Table 1, HR: 2.32, *p* = 1.9e−05). The subgroup analysis also showed that the prediction impact of IRGPI on OS of metastatic melanoma was promising in training cohort (Table 3).

**Table 3:** The results of clinical subgroup analysis of prognosis based on the IRGPI.

Level	Number of patients		HR (95%CI)	Log rank *p* value
	Low IRGPI risk	High IRGPI risk		
**All**				
	192	162	3.189 (2.342–4343)	4.35E−17
**Age**				
< 60	116	86	3.197 (2.063–4.955)	4.73E + 210
≥ 60	76	76	2.97 (1.924–4.585)	1.732–07
**Gender**				
Male	111	113	2.897 (2.017–4.162)	4.492–10
Female	81	49	3.683 (2.031–6.679)	2.572–08
**T stage**				
T0	19	4	8.89 (0.516–153.279)	0.0002
T1	25	15	4.462 (1.167–17.058)	0.0019
T2	44	27	3.622 (1.709–7.678)	8.34E−06
T3	39	41	2.603 (1.433–4.73)	0.0007
T4	28	40	2.465 (1.345–4.517)	0.0032
**N stage**				
N0	92	79	2.758 (1.814–4.193)	7.20E−08
N1	36	29	3.731 (1.762–7.899)	0.0001
N2	23	16	4.817 (1.663–13.957)	0.0005
N3	22	23	2.864 (1.21–6.776)	0.0109
**M stage**				
M0	171	142	3.083 (2.235–4.254)	1.04E−14
M1	8	11	4.612 (0.977–21.764)	0.0318
**Stage**				
I/II	73	60	3.071 (1.846–5.11)	1.21E−07
III/IV	86	75	3.72 (2.304–6.005)	1.85E−09
**Radiation therapy**				
NO	141	123	3.633 (2.56–5.155)	1.86E−16
YES	43	29	2.049 (1.046–4.013)	0.0195

Abbreviation: *HR* hazard ratio, *CI* confidential interval, *IRGPI* immune-related gene pairs index.

**Figure 4:**
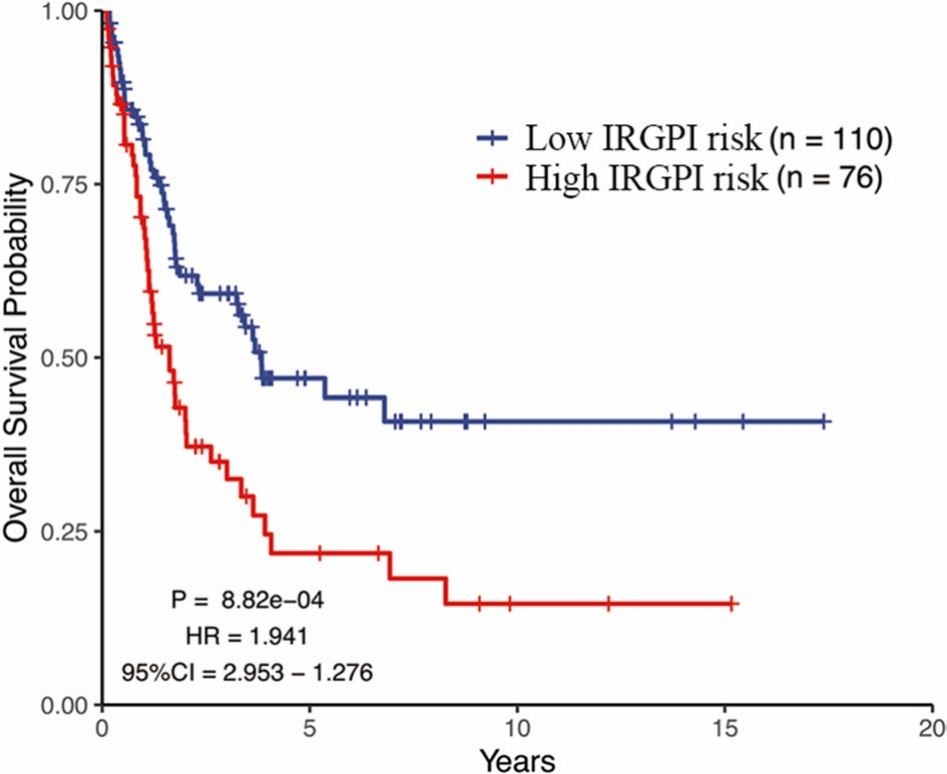
Kaplan**–**Meier curves analysis among different IRGPI risk groups in testing cohort. It showed that the impact of IRGPI on overall survival in testing cohort.

The IRGPI was also performed into the independent GSE65904 testing cohort to validate its accuracy and prediction ability. The patients of testing cohort were divided into high and low-IRGPI risk group based on the same cut off. The KM curve analysis indicated that patients with the high-IRGPI risk also had poor OS compared with the low-IRGPI risk group (Fig. 4, HR:1.914, 95%CI: 1.276–2.953, *p* = 8.82e−04).

### Different IRGPI risk groups displayed differential tumor microenvironment

**Figure 5:**
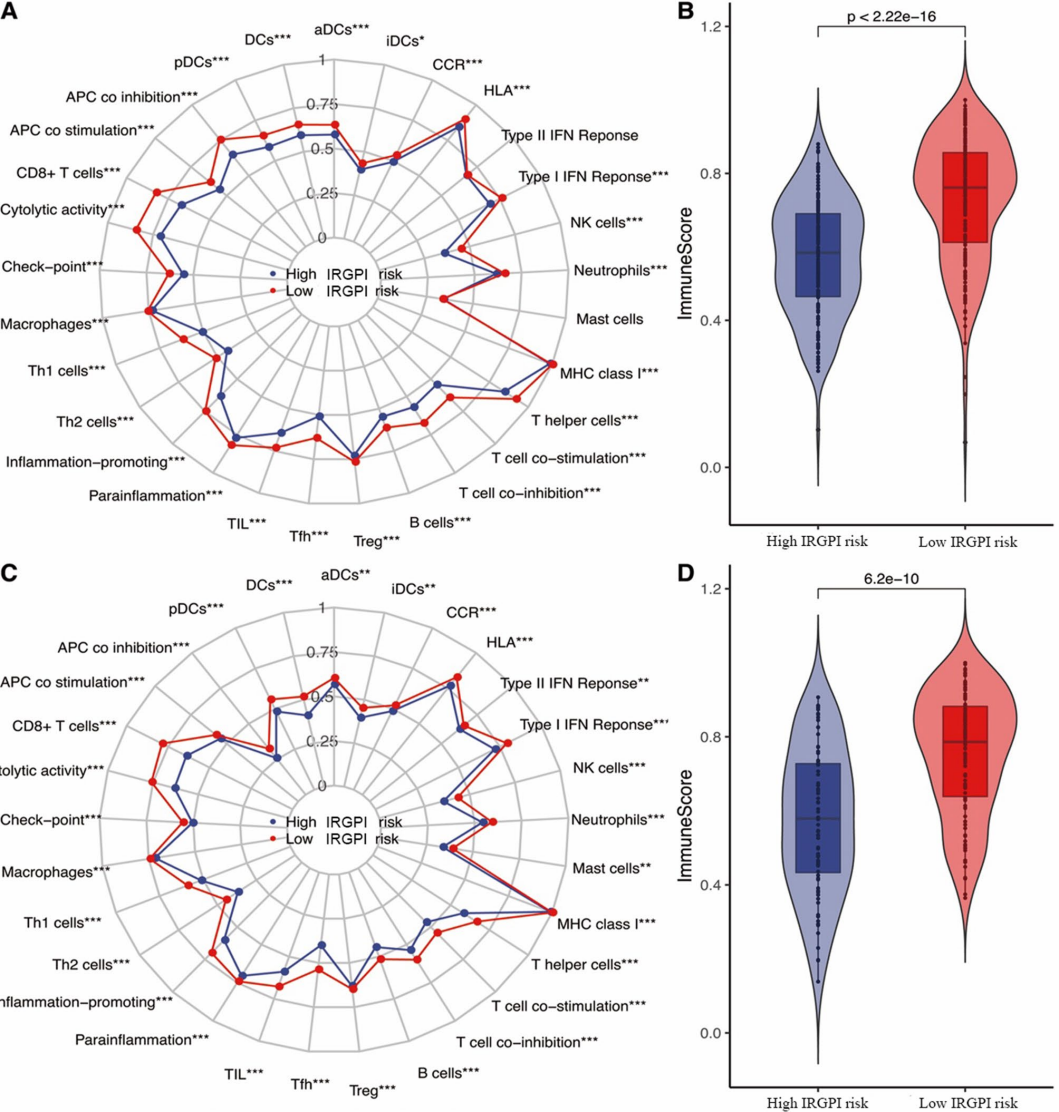
Tumor immune microenvironment status within differential immune risk groups by using ssGSEA. (**A**) Summary of the 29 immune cells score of differential immune risk groups in training cohort. (**B**) The total immune score of differential immune risk groups in training cohort. (**C**) Summary of the 29 immune cells score of differential immune risk groups in testing cohort. (**D**) The total immune score of differential immune risk groups in testing cohort. All *p* values were estimated by t test (*p < 0.05, **p < 0.01, ***p < 0.001).

The relative scores of 29 immune cells for each patient were estimated by ssGSEA algorithm. The comparative summary of ssGSEA output result was performed in these two risk groups and a wide variety of differential immune infiltration cells existed. Moreover, both the training cohort and the testing cohort showed the low-IRGPI risk group had a better total immune score than the high-IRGPI risk group by ESTIMATE algorithm (Fig. 5B , *p* = 2.22e−16; Fig. 5D , *p* = 6.2e−10).

### Immune related biological processes in different IRGPI risk groups

**Figure 6:**
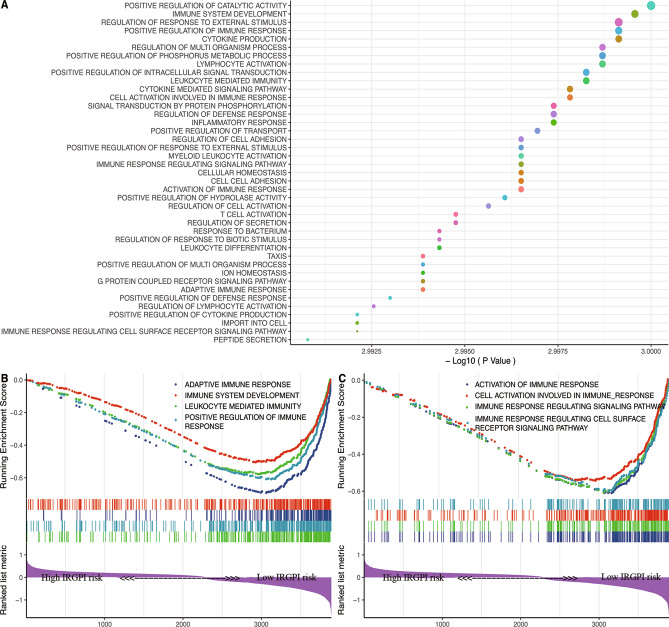
Parts of Gene Set Enrichment Analysis (GSEA) results, including adaptive immune response; immune system development; leukocyte mediated immunity; positive regulation of immune response; activation of immune response; cell activation involved in immune response; immune response regulating signaling pathway; immune response regulating cell surface receptor signaling pathway.

In GSEA analysis, various immune-associated biological processes were enriched, such as adaptive immune response; immune system development; leukocyte mediated immunity; positive regulation of immune response; activation of immune response; cell activation involved in immune response; immune response regulating signaling pathway; immune response regulating cell surface receptor signaling pathway (Fig. 6).

## Discussion

With the development of researches on tumor immunity microenvironment, immunotherapy has been listed as a successful treatment option for a wide variety of cancers, including metastatic melanoma^26,27^. As an emerging and effective therapeutic approach, tumor immunotherapy has a broad prospect in metastatic melanoma^14,16,28^. In our study, we defined 376 IRGs and 119 IRGPs that were closely related to metastatic melanoma, which would provide us with powerful conditions to establish a novel prognosis model for patients with metastatic melanoma based on immunogenomic landscape analysis.

Through further analysis, 24 IRGPs, including 45 unique IRGs, were obtained, and the IRGPI of metastatic melanoma was further established based on the 24 IRGPs. The optimal cut off for distinguishing patients into high and low-IRGPI risk groups was identified based on the time-dependent ROC curve within five years. Further, KM curve analysis was used to examined the effect of IRGPI on the prognosis prediction of metastatic melanoma, and the results showed that the patients with high-IRGPI risk were associated with worse OS and DFS, indicating a poor prognosis in high-IRGPI risk group. Meanwhile, we also proved that IRGPI could be used as a promising and independent prognostic model by multivariate Cox analysis and subgroup analysis. Moreover, we used immune gene pairs for data analysis, which would not consider the technical deviation of different platforms to the greatest extent, and successfully solved the problem of different data platforms for expression. This would bring great hope for more accurate prognosis prediction in metastatic melanoma. Therefore, it is evident that IRGPI has a promising potential to be employed as a reliable prognostic index for metastatic melanoma.

The IRGPI prognosis index consisted of 45 unique IRGs, and many of them have been shown to be strongly associated with cancers development. The latest research reported that *IRF-1* could be used as an indicator of PD-L1 expression capability in anti-PD-1 therapy, so it could predict the therapeutic effect of metastatic melanoma^29^. Similarly, *IDO-1* was also reported that was closely related to the anti-PD-1 response of metastatic melanoma^30^. Reynders et al. described two variants of *CXCR3*, including *CXCR3-A* and *CXCR3-B*, played opposite cellular roles in cancer. Among them, *CXCR3-A* involved in cell dissemination and proliferation through G protein signaling pathway, while *CXCR3-B* could inhibit cell migration, proliferation and induce cell apoptosis^31^. Wente et al. proved that the expression of *CXCL14* in pancreatic cancer was significantly higher than normal pancreatic tissue, especially in the frontier tissue of invasive pancreatic cancer, which indicated that *CXCL14* could play an important role in tumor metastasis^32^. Additionally, related researches showed the over-expression of *ITGAV* was closely related to the development of colorectal cancer and spreading of colorectal cancer cells via perineural invasion^33^. And the high expression of *PIK3CD* was also proved that affected the distant metastasis and poor prognosis of colon cancer^34^. These studies indicated that the IRGs in IRGPI played an important role in the occurrence and development of cancers, suggesting that our prognostic model would have good prospect and prognostic value in metastatic melanoma.

The infiltration of immune cells in the tumor microenvironment played an important role in the progression of cancers, and it was also reported in metastatic melanoma in a wide variety studies^27,35^. Therefore, we calculated the score of 29 immune cells infiltration in each sample using ssGSEA to further explore the correlation between tumor microenvironment immune cells infiltration and IRGPI. The results showed that different IRGPI risk groups displayed differentiates tumor immunity microenvironment. The immune infiltration cells played different roles in metastatic melanoma. For example, CD8+TIL has been reported for the treatment of refractory metastatic melanoma patients and obtained approved curative effect^36^. Romero et al. found that the increase of CD4+NKG2D+Th1 in patients with metastatic melanoma was associated with prolonged survival^37^. Another study suggested that IL-2 treatment could restore Th1 ⁄ Th2 balance in metastatic melanoma and activate lymphocytes. At the same time, it enhanced the ability of monocytes producing IFN-γ, which induced a systemic immune response, thus obtained improved prognosis and better clinical benefits^38^. This is consistent with our results. Furthermore, in the pulmonary metastasis of melanoma in mice, Saga et al. proved that local generated melanoma-specific CTLs could significantly reduce the number of metastatic foci^39^. These studies confirmed that immune cells differential infiltration could be a potential mechanism of IRGPI prognosis prediction role, but these observations could be further explored to fully understand the subtle differences in microenvironment immune cell infiltration. The results strengthened our understanding of immune cells infiltration in the metastatic melanoma tumor microenvironment, which provided the conditions for further exploration.

We conducted GSEA to explore the biological processes of IRGPI acting on metastatic melanoma and demonstrated that large number of immune-related processes were differentially enriched between high and low IRGPI risk groups. Existing studies have shown that positive regulation of the immune response could reduce the risk of death from lymphatic metastasis of melanoma, and it was closely related to the increase and activation of tumor infiltrating lymphocytes (TIL)^40^. Immune response has shown to be involved in tumor development by modulating cell surface receptor signaling pathways. For example, the dynamic variations of the cell surface receptor C-X-C Motif Chemokine Receptor 3 (CXCR3) played an important role in the metastasis of melanoma^41^. Leukocyte mediated immunity could inhibit the metastasis of melanoma, such as cytotoxic T cell (CTL), which could reduce the metastatic foci^39^. This was consistent with the previous understanding. In addition, the immune response regulating signaling pathway may play a role in tumor progression and metastasis through the regulation of PD-L1 expression^42^. These results suggested that immune-related signaling pathways were an important aspect in affecting tumor progression. Our study elucidated the relevance of IRGPI in multiple immune biological processes, providing theoretical support for the application of IRGPI and the immune-related studies of subsequent metastatic melanoma.

## Conclusion

The study breaks the limitations of traditional studies and reduces the biological heterogeneity and technical bias of different cross-sequencing platforms through the application of the gene pairs, which provided great convenience and reliability for the establishment of the prognostic index. Although our research showed advantages in data processing, there were still some limitations to consider. First, due to the lack of in vitro or in vivo experiments, the reliability of our molecular mechanism analysis results was limited. Second, this study was a retrospective study and a well-designed clinical trial was needed to further verify our results, though the mechanism of action of IRGPI in metastatic melanoma has been effectively elucidated through multiple methods. Therefore, the findings would provide new insights into the clinical decision-making and prognostic monitoring of metastatic melanoma and provide theoretical support for further researches.

## Data Availability

R 3.6.3 (http://www.r-project.org/) is an open source software. The RNA-FPKM data and clinical data of melanoma samples are obtained from the TCGA data portal (https://cancergenome.nih.gov/). The expression profiles of GSE65904 are downloaded through GEOquery R package. 1811 unique immune-related genes (IRGs) are acquired from ImmPort database (https://immport.niaid.nih.gov). The c5.bp.v7.1.entrez.gmt file come from Molecular Signatures Database (MSigDB, http://software.broadinstitute.org/gsea/index.jsp).

## Abbreviations

IRGs: Immune-related genes
IRGPs: Immune-related gene pairs
IRGPI: Immune-related gene index
MAD: Median absolute deviation
ROC: Receiver operating characteristic
KM: Kaplan**–**Meier
TCGA: The cancer genome atlas
GEO: Gene expression omnibus
FPKM: Fragments per kilobase of exon model per million mapped reads
FDR: False discovery rate
AUC: Area under curve
ssGSEA: Single-sample gene-set enrichment analysis
GSEA: Gene set enrichment analysis
MSigDB: Molecular signatures database
OS: Overall survival
DFS: Disease-free survival
